# Supplementary material for: Antioxidant Capacity and Protective Effects on H2O2-Induced Oxidative Damage in PC12 Cells of the Active Fraction of Brassica rapa L
Source: Foods. 2023 May 22;12(10):2075. doi: 10.3390/foods12102075 (PMC10217163; doi:10.3390/foods12102075)
Supplement: Supplementary file 1 [file foods-12-02075-s001.zip › foods-2376672-supplementary.pdf]

Table S1. Compounds detected by UPLC-MS of BREE-Ea.

| Compounds | M/Z | Formula | RT | Type | Mode | Relative Peak Area | Functions | Combined targets related with neurodegenerative diseases |
|---|---|---|---|---|---|---|---|---|
| Oleoside dimethyl ester | 419.15 | $C_{18}H_{26}O_{11}$ | 170.46 | terpenoids | POS | 4680352.80 | Treatment of osteoporosis [57]. | Neurodegenerative diseases. |
| 2-(acetylamino)-1,5-anhydro-2-deoxy-4-O-b-D-galactopyranosyl-D-arabino-Hex-1-enitol | 366.14 | $C_{14}H_{23}NO_{10}$ | 366.12 | other | POS | 35974526.78 | Metabolomic markers for predicting preeclampsia in early pregnancy [58]. | N/A. |
| Phosphoric acid | 98.98 | $H_3O_4P$ | 571.44 | other | POS | 155714.94 | Metabolic processes. | N/A. |
| Tyrosyl-Cysteine | 285.09 | $C_{12}H_{16}N_2O_4S$ | 400.02 | amino acids | POS | 12543583.84 | Potential immunomodulatory substances [59]. | N/A. |
| L-Tryptophan | 243.05 | $C_{11}H1_1KN_2O_2$ | 121.75 | amino acids | POS | 574589.32 | _Metabolism in immune regulation [60]. | Neurodegenerative diseases. |
| Rotundine B | 234.19 | C15H23NO | 342.94 | alkaloids | POS | 169955637.80 | Potential biomarkers [61]. | N/A. |
| Pterosin H | 251.13 | $C_{15}H_{19}ClO$ | 337.43 | other | POS | 27995976.17 | N/A. | N/A. |
| Heliannuol C | 249.15 | $C_{15}H_{20}O_3$ | 393.77 | terpenoids | POS | 17215256.05 | Inhibited the growth of etiolated wheat coleoptiles [62]. | N/A. |
| Bergenin | 329.08 | $C_{14}H_{16}O_9$ | 114.65 | polyphenol s | POS | 3696260.27 | Anti-inflammation [63]. | Neurodegenerative diseases. |

| | | | | | | | | |
|---|---|---|---|---|---|---|---|---|
| Nobiletin | 403.14 | $C_{21}H_{22}O_8$ | 456.75 | flavonoid | POS | 7538036.14 | Antioxidant, anti-AD, anti-inflammatory, anti-cancer, cholesterol lowering and memory protection activities [53,64-65]. | Neurodegenerative diseases. |
| Tangeritin | 373.13 | $C_{20}H_{20}O_7$ | 480.70 | flavonoid | POS | 13455714.00 | Anti-inflammatory, antioxidant, and protect the nerves [66, 67]. | Neurodegenerative diseases. |
| Linoleoyl ethanolamide | 324.29 | $C_{20}H_{37}NO_2$ | 599.24 | alkaloids | POS | 484132943.00 | Anti-inflammatory [68]. | N/A. |
| Melilotocarpan C | 331.12 | $C_{18}H_{18}O_6$ | 434.71 | flavonoid | POS | 10929394.95 | Antiplasmodial activity [69]. | N/A. |
| 4-Isothiocyanato-1-butene | 114.04 | $C_5H_7NS$ | 317.62 | other | POS | 66596292.65 | Aromatic [70]. | N/A. |
| (R)-(E)-Sulforaphene | 176.02 | $C_6H_9NOS_2$ | 381.73 | alkaloids | POS | 6274769.66 | Anticancer [71]. | N/A. |
| Isorhamnetin | 317.07 | $C_{16}H_{12}O_7$ | 314.53 | flavonoid | POS | 87861587.20 | Protect cardiovascular and cerebrovascular, anti-tumor, anti-inflammatory, antioxidant, organ protection, prevention of obesity [72]. | Neurodegenerative diseases. |
| Dioscoretine | 242.18 | $C_{13}H_{23}NO_3$ | 348.42 | alkaloids | POS | 1761177.53 | Lipid metabolites [73]. | |
| Cycloalliin | 178.05 | $C_6H_{11}NO_3S$ | 265.18 | flavonoid | POS | 35787524.81 | Serum TG-lowering [74]. | N/A. |
| N-Phenyl-2-naphthylamine | 220.11 | $C_{16}H_{13}N$ | 557.46 | alkaloids | POS | 11801613.04 | A suspected carcinogen [75]. | Neurodegenerative diseases. |
| 4-Aminophenol | 110.06 | $C_6H_7NO$ | 124.85 | other | POS | 81913701.29 | N/A. | N/A. |

| | | | | | | | | |
|---|---|---|---|---|---|---|---|---|
| 5,7-Dihydroxy-6-methoxyflavone 5-rhamnoside | 431.13 | $C_{22}H_{22}O_9$ | 171.63 | flavonoid | POS | 8195352.06 | Anti-inflammatory and anti-aging [76]. | N/A. |
| Solavetivone | 219.17 | $C_{15}H_{22}O$ | 456.75 | terpenoids | POS | 7095735.20 | Phytoalexins [77]. | N/A. |
| Kanokoside A | 477.19 | $C_{21}H_{32}O_{12}$ | 521.73 | other | POS | 5064455.48 | N/A | N/A. |
| Cabbage identification factor 2 | 329.07 | $C_{15}H_{12}N_4O_3S$ | 279.89 | terpenoids | POS | 157596669.40 | N/A | N/A. |
| Biotin | 245.10 | $C_{10}H_{16}N_2O_3S$ | 177.29 | other | POS | 112622741.30 | It is essential for human health, growth and development [78]. | N/A. |
| Cyperotundone | 219.17 | $C_{15}H_{22}O$ | 389.98 | terpenoids | POS | 61454993.32 | Anti-breast cancer [79]. | N/A. |
| Coniferin | 365.12 | $C_{16}H_{22}O_8$ | 313.47 | other | POS | 57294928.98 | N/A | Neurodegenerative diseases. |
| Linatine | 260.13 | $C_{10}H_{17}N_3O_5$ | 326.44 | amino acids | POS | 37833388.00 | Vitamin B6 antagonist [80]. | N/A. |
| 4-Methoxycinnamoyloleanolic acid methyl ester | 631.45 | $C_{41}H_{58}O_5$ | 652.37 | terpenoids | POS | 25460431.39 | N/A | N/A. |
| 2-Acetoxy-3-geranylgeranyl-1,4-dihydroxybenzene | 441.30 | $C_{28}H_{40}O_4$ | 755.16 | terpenoids | POS | 21852900.58 | N/A | N/A. |
| Dihydroneopterin phosphate | 336.07 | $C_9H_{14}N_5O_7P$ | 205.35 | alkaloids | POS | 20369023.77 | N/A | N/A. |

| | | | | | | | | |
|---|---|---|---|---|---|---|---|---|
| 3-Mercapto-2-methylpentanal | 133.07 | $C_6H_{12}OS$ | 348.42 | terpenoids | POS | 16366353.60 | N/A | N/A. |
| Ceanothenic acid | 455.31 | $C_{29}H_{42}O_4$ | 777.47 | terpenoids | POS | 770317.84 | Antibacterial activity [81]. | N/A. |
| (R)-Oxypeucedanin | 287.09 | $C_{16}H_{14}O_5$ | 329.88 | other | POS | 175976401.00 | A protective effect against apoptosis induced by DOX in PC12 cells by inhibition of ROS production [82]. | N/A. |
| Verimol A | 317.14 | $C_{18}H_{20}O_5$ | 426.60 | organic acids | POS | 132888419.70 | N/A. | N/A. |
| Arborinine | 286.10 | $C_{16}H_{15}NO_4$ | 440.12 | alkaloids | POS | 673115.81 | Against Cancer [83]. | N/A. |
| (Z)-Resveratrol | 229.09 | $C_{14}H_{12}O_3$ | 315.18 | polyphenol s | POS | 54477858.00 | Anti-oxidation, anti-AD, antibacterial, anticancer [56, 84-85]. | Neurodegenerative diseases. |
| 5-(2-Hydroxyethyl)-4-methylthiazole | 144.05 | $C_6H_9NOS$ | 251.33 | alkaloids | POS | 25263028.79 | Involved in the synthesis of thiamin and thiamin diphosphate in yeasts [86]. | N/A. |
| Ginsenoside Rh5 | 653.46 | $C_{37}H_{64}O_9$ | 651.14 | other | POS | 18901669.27 | N/A | N/A. |
| Sulfolithocholic acid | 457.27 | $C_{24}H_{40}O_6S$ | 762.28 | other | POS | 47504212.89 | Maintain intestinal homeostasis [87]. | N/A. |

| | | | | | | | | |
|---|---|---|---|---|---|---|---|---|
| Dihydrogenistein | 273.07 | $C_{15}H_{12}O_5$ | 289.39 | flavonoid | POS | 278423487.10 | N/A | N/A. |
| Cucurbitachrome 1 | 601.44 | $C_{40}H_{56}O_4$ | 735.77 | other | POS | 1375864136.00 | N/A | N/A. |
| Isoleucyl-Aspartate | 247.13 | $C_{10}H_{18}N_2O_5$ | 340.73 | amino acids | POS | 743083378.30 | N/A | N/A. |
| Anofinic acid | 205.09 | $C_{12}H_{12}O_3$ | 585.05 | organic acids | POS | 584564520.30 | A plant pathogenic fungus, against Cladosporium cucumerinum [88]. | N/A. |
| Verimol C | 301.14 | $C_{18}H_{20}O_4$ | 585.05 | other | POS | 495568335.10 | N/A | N/A. |
| Arachidonic acid | 305.25 | $C_{20}H_{32}O_2$ | 614.38 | organic acids | POS | 399982534.60 | Arachidonic acid, anti-tumor [89,90]. | Neurodegenerative diseases. |
| Diethyl phthalic acid | 221.08 | $C_{12}H_{14}O_4$ | 338.08 | organic acids | NEG | 67107484.18 | N/A. | N/A. |
| m-Coumaric acid | 163.04 | $C_9H_8O_3$ | 277.02 | organic acids | NEG | 4680352.80 | Antioxidant [91]. | Neurodegenerative diseases. |
| Abscisic acid | 263.13 | $C_{15}H_{20}O_4$ | 340.89 | organic acids | NEG | 27587783.73 | Phytohormone. | N/A. |
| 2,4-Dihydroxybenzoic acid | 153.02 | $C_7H_6O_4$ | 136.79 | organic acids | NEG | 155714.94 | Food additive [92]. | N/A. |
| Porphobilinogen | 235.05 | $C_{10}H_{14}N_2O_4$ | 263.95 | alkaloids | NEG | 64133491.92 | Screening for porphyria [93]. | N/A. |
| Indole-3-carbinol | 146.06 | $C_9H_9NO$ | 332.39 | alkaloids | NEG | 16294984.77 | Anti-tumor [94]. | N/A. |

| | | | | | | | | |
|---|---|---|---|---|---|---|---|---|
| 3,4-Dihydroxy-trans-cinnamate | 179.03 | $C_9H_8O_4$ | 248.59 | polyphenols | NEG | 12543583.84 | Antioxidant [95]. | N/A. |
| Undecylenic acid | 183.14 | $C_{11}H_{20}O_2$ | 367.45 | organic acids | NEG | 574589.32 | Anti-tumor, inhibit phenoloxidase [96, 97]. | N/A. |
| Dihydrojasmonic acid | 211.13 | $C_{12}H_{20}O_3$ | 596.17 | organic acids | NEG | 169955637.80 | A plant growth regulator [98]. | N/A. |
| 4-Hydroxystyrene | 119.05 | $C_8H_8O$ | 309.66 | polyphenols | NEG | 27995976.17 | N/A. | N/A. |
| [6]-Dehydrogingerdione | 289.14 | $C_{17}H_{22}O_4$ | 466.28 | polyphenols | NEG | 15710884.63 | Enhancements of skin cell proliferations and migrations, anti-inflammation, neuroprotective effect [99-101]. | N/A. |
| Myristoleic acid | 225.19 | $C_{14}H_{26}O_2$ | 614.27 | organic acids | NEG | 14331249.80 | Inhibits osteoclast formation and bone resorption, reduce obesity [102, 103]. | N/A. |
| Hexadecanedioic acid | 285.21 | $C_{16}H_{30}O_4$ | 423.05 | organic acids | NEG | 3696260.27 | N/A. | N/A. |
| Phthalic acid | 165.02 | $C_8H_6O_4$ | 91.77 | organic acids | NEG | 13693837.22 | It has been widely used in the preparation of phthalate plasticizer [104]. | Neurodegenerative diseases. |

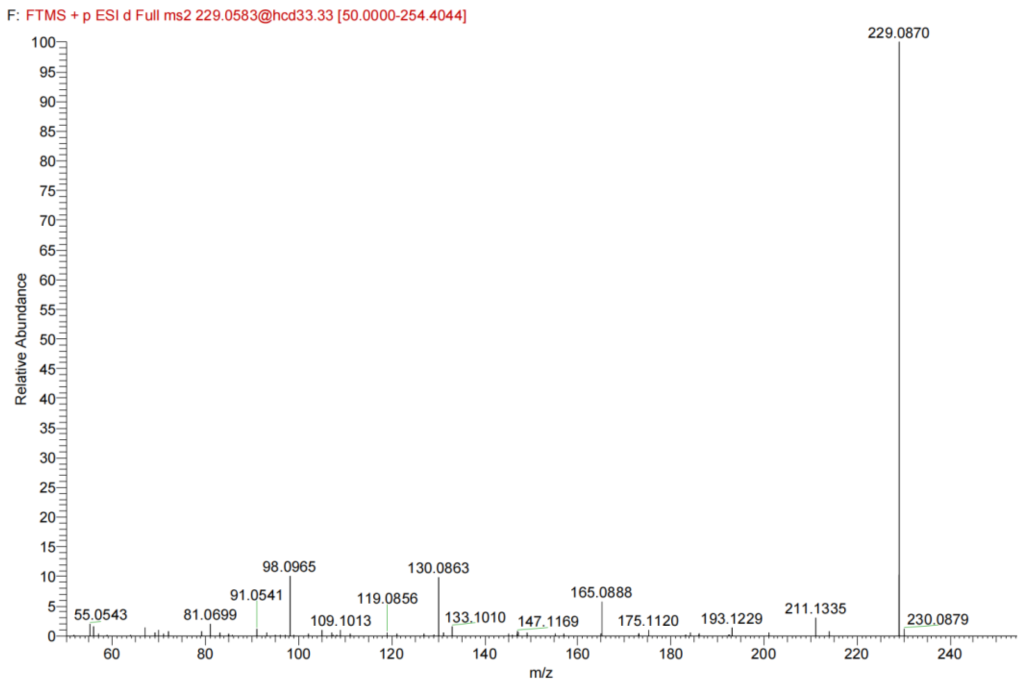


Figure S1. Mass spectrum in (Z)-Resveratrol. RT: 5.24.

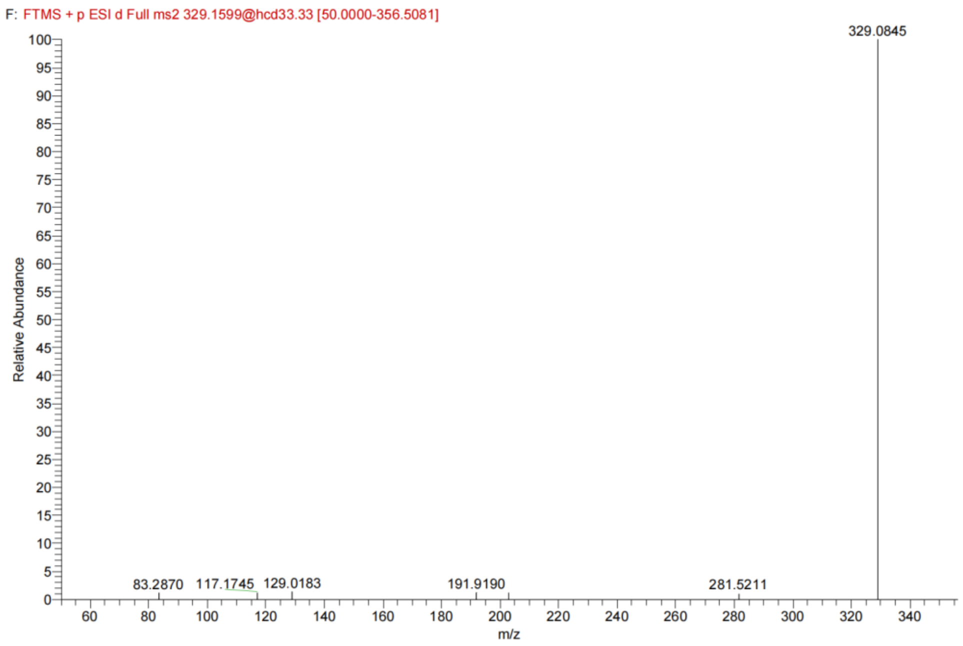


Figure S2. Mass spectrum in Bergenin. RT: 1.92.

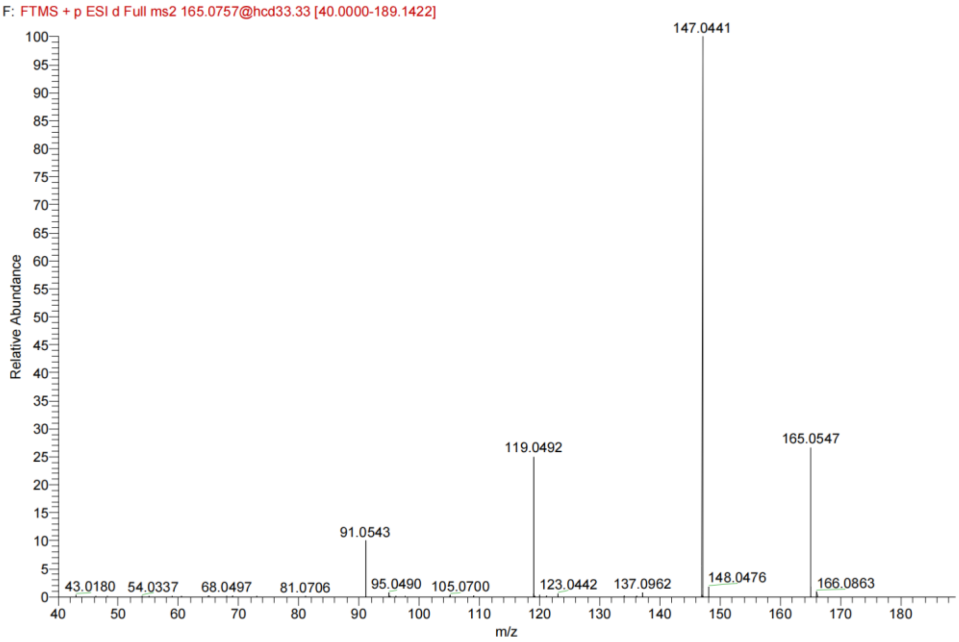


Figure S3. Mass spectrum in m-Coumaric acid. RT: 4.30.

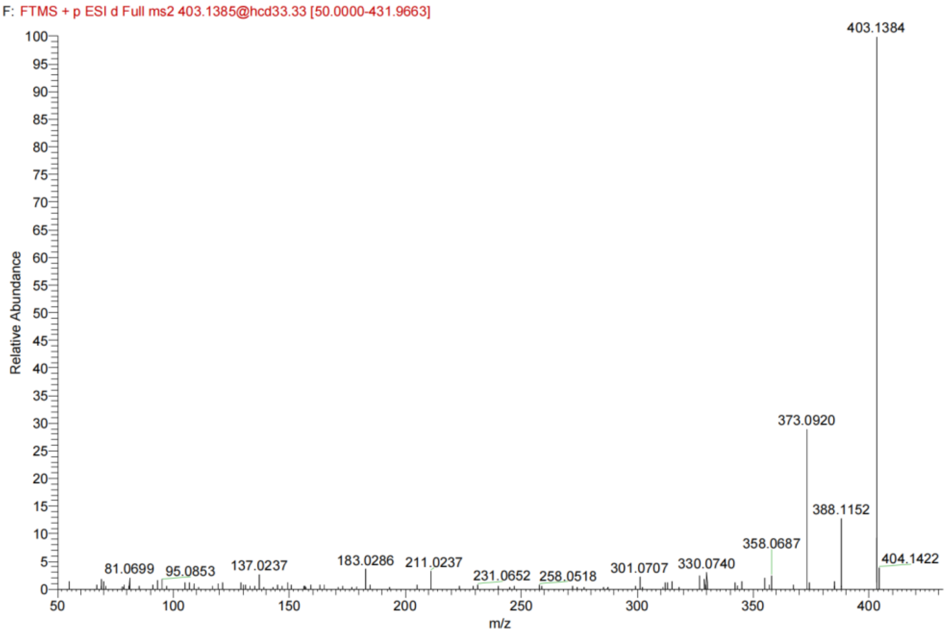


Figure S4. Mass spectrum in Nobiletin. RT: 7.62.

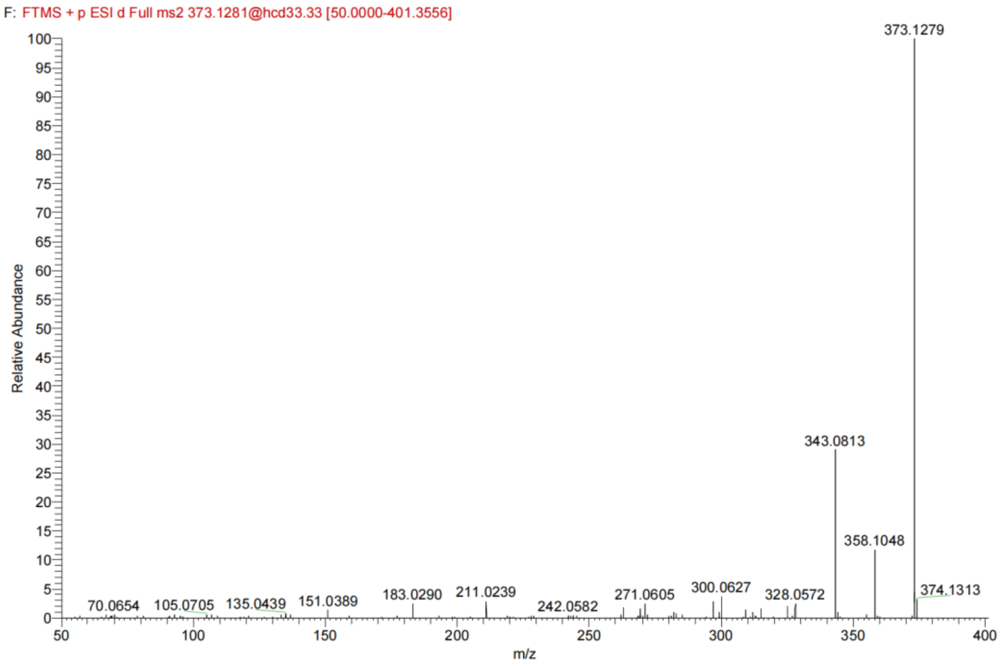


Figure S5. Mass spectrum in Tangeritin. RT: 8.00.

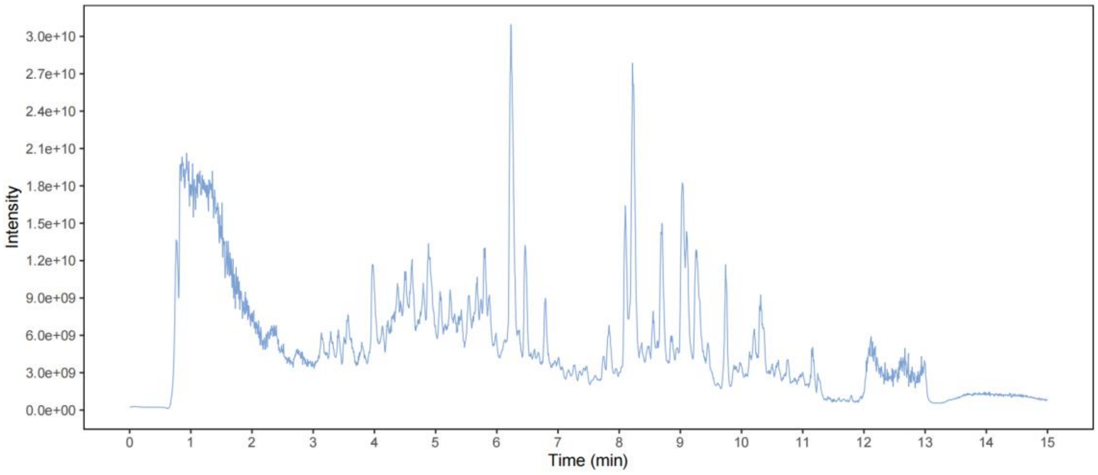


Figure S6. Total ion maps of compounds from *Brassica rapa* L. under positive ion model.

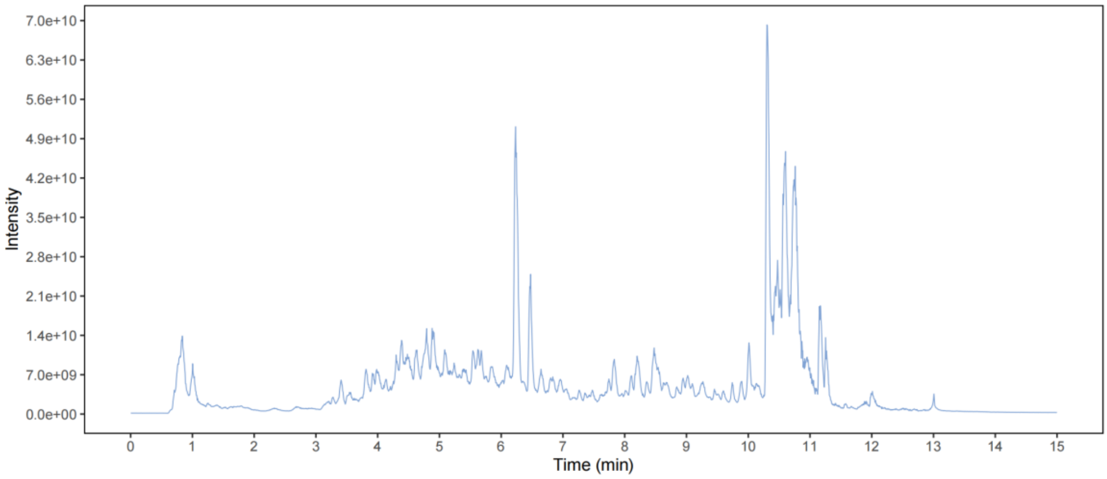


Figure S7. Total ion maps of compounds from *Brassica rapa* L. under negative ion model.
